# ANRIL rs1333049 C/G polymorphism and coronary artery disease in a North Indian population - Gender and age specific associations

**DOI:** 10.1590/1678-4685-GMB-2019-0024

**Published:** 2020-03-16

**Authors:** Naindeep Kaur, Jagtar Singh, Sreenivas Reddy

**Affiliations:** 1 Department of Biotechnology, Panjab University, Chandigarh, India.; 2 Department of Cardiology, Postgraduate Institute of Medical Education and Research, Chandigarh, India.

**Keywords:** Genetic polymorphism, Coronary artery disease, North Indian Population, ARMS-PCR, epidemiology study

## Abstract

Many studies conducted worldwide substantiate a role of genetic polymorphisms in non-coding regions linked with coronary artery disease (CAD). One such single nucleotide polymorphism (SNP) of a non-coding RNA in the INK4 locus (ANRIL) *i.e.* rs1333049 C/G in the vicinity of cell cycle regulating genes is documented to have a role in CAD risk. In this study we aimed to determine the association of ANRIL rs1333049 C/G with CAD in a North Indian population. Five hundred disease free controls and 500 CAD patients were genotyped using allele specific ARMS-PCR method. High risk association of rs1333049 was seen in both heterozygous and mutant genotypes (OR=2.883, 95% CI=1.475-5.638 and p=0.002 and OR=6.717, 95% CI=3.444-13.102 and p < 0.001 respectively). Gender stratified analysis revealed risk association in both heterozygous and mutant genotypes in males. However, risk association in the mutant genotype and females was documented. Similarly, risk association was seen in subjects above 40 years of age in heterozygous and mutant genotypes. Similarly, risk association was reported in obese, sedentary lifestyle, positive family history and smoking in the heterozygous and mutant genotype and with diabetes in the mutant GG genotype. The study revealed high risk association of ANRIL rs1333049 with CAD and other risk factors.

## Introduction

Coronary artery disease (CAD) has become epidemic worldwide and a major barrier to sustainable human development. It has been lately observed that around 16.5 million people above 20 years of age in United States of America (U.S.) suffer from CAD. Not only that, the prevalence increases in both genders with a gradual increase in age (Sanchis-Gomar *et al.*, 2016). The incidence in developing countries like India is also alarming and studies have reported a boost in CAD prevalence since past half century. There are a number of established key modifiable and non-modifiable factors like age, gender, genetics, smoking, dyslipidemia, hypertension, diabetes, obesity, high-fat diet, physical inactivity, drug abuse, alcohol consumption and mental stress attributing significant risk towards the disease.

An individual’s risk of harboring CAD is inflected by the interplay between genetic and lifestyle factors established by the multifactorial nature of CAD. A genetic component in CAD is validated from the increased risk in first degree relatives of the affected individuals, high lifetime risk in the offspring if parents are affected and high concordance in monozygotic than dizygotic twins. The first Genome Wide Association (GWA) studies for CAD were published in 2007 and since then, a number of genetic variants at various chromosomal loci specific to CAD in various populations have been identified (Scheffold *et al.*, 2011).

GWA studies document a locus on chromosome 9p21 linked to CAD (Jarinova *et al.*, 2009; Cunnington *et al.*, 2010; Ahmed *et al.*, 2013). Though this 58 kb locus lacks the genes associated with atherosclerosis, an *antisense non-coding RNA in the INK4 locus (ANRIL)* gene dwells within the vicinity of cell cycle regulating genes in this region. It is reported to be in strong linkage disequilibrium with cell cycle proliferatory genes such as *cyclin dependant kinase inhibitors 2A and 2B (CDKN2A and CDKN2B)* (Cunnington *et al.*, 2010). *CDKN2A* is basically a tumor suppressor gene and encodes two proteins *viz.* p14ARF and p16. p16 controls the G1 to S transition in the cell cycle and p14ARF stimulates cell cycle arrest in G2 phase, subsequently leading to cell death. *CDKN2B* lies adjoining the *CDKN2A* and encodes proteins that inhibit the cell cycle G1 progression (Cunnington *et al.*, 2010).

*CDKN2B anti-sense RNA (CDKN2B-AS1)* spans about 126.3 kb and overlaps with *CDKN2B (p15)* at the 5’ end and comprises of 20 exons that are prone to alternative splicing (Jarinova *et al.*, 2009) and reported to be linked to CAD risk (Matsuoka *et al.*, 2015; Dehghan *et al.*, 2016), hypertension (Bayoglu *et al.*, 2016) and stroke (Bai *et al.*, 2014). CDKN2B-AS or CDKN2B-AS1 or INK4 are used as synonyms for ANRIL. The *ANRIL* locus is reported to alter the expression of neighbouring genes by apparently acting either by chromatin remodeling, DNA methylation, gene silencing or RNA interference (Jarinova *et al.*, 2009).

The SNP rs1333049 C/G is positioned in the 3’UTR (untranslated region) of *CDKN2B-AS1* and considered to have a crucial role in advancement of cardio and cerebro-vascular disease by modifying dynamics of vascular smooth muscle cell proliferation (Cunnington *et al.*, 2010). The Wellcome Trust Case Control consortium study has documented rs1333049 as displaying powerful association with CAD (Consortium, 2007). The association of rs1333049 was studied in CAD (Dechamethakun *et al.*, 2014; Haslacher *et al.*, 2016), atherosclerosis (Bochenek *et al.*, 2013) and Alzheimer’s disease (Popov *et al.*, 2010).

The present study was conducted with the aim of determining allelic and genotypic frequencies of *ANRIL* rs1333049 and risk association with CAD and other selected parameters in a North Indian population.

### Material and Methods

#### Study population

One thousand individuals aged 25-70 years of both sexes were enrolled to evaluate the role of *LOX1* rs11053646 G/C and rs1050283 C/T polymorphisms in CAD. Five hundred patients belonging to North Indian states (Jammu and Kashmir, Haryana, Chandigarh, Punjab, Himachal Pradesh, New Delhi, Uttaranchal, Uttar Pradesh, Uttarakhand and Rajasthan) visiting the Department of Cardiology at Postgraduate Institute of Medical Education and Research, Chandigarh and documented CAD on coronary angiogram with more than 50% stenosis in at least one epicardial coronary artery) were registered as cases. Subjects with acute/chronic infection, hepatic dysfunction, renal dysfunction, severe heart failure, hypo or hyperthyroidism, pregnancy and malignancy were excluded. Five hundred healthy individuals satisfying the inclusion criteria (with absence of any cardiac disorder, chronic diseases such as diabetes, hypertension, hypo- or hyperthyroidism, tuberculosis, hepatitis, AIDS, malignancy and pregnancy) were enrolled as controls*.* Subjects with history of smoking, alcohol consumption and tobacco chewing were also excluded. Majority of the controls were donors at the blood donation camps. A written informed consent was given by all participants prior to enrollment. The study was approved by the Institutional Ethics Committee, Panjab University, Chandigarh, India and performed according to the “Ethical Guidelines for Biomedical Research on Human Participants, 2006” as proposed by the Indian Council of Medical Research and Ministry of Health, Govt. of India.

#### Biometric and biochemical measurements

Anthropometric parameters like height, weight, waist to hip ratio, BMI and blood pressure were noted. Risk factors for CAD like diabetes, hypertension, dyslipidemia, family history, smoking and drinking habits were recorded. Lipid profile, fasting blood glucose, hsCRP, uric acid Apolipoprotein A1 and Apolipoprotein B determination was done by standard biochemical methods.

#### DNA isolation, SNP selection and genotyping

Five milliliters of venous blood sample was collected in EDTA-coated vials and DNA was isolated by the sodium saline citrate buffer method (Roe *et al.*, 1996). Genotyping of the *ANRIL* rs1333049 C/G polymorphism was done by allele-specific ARMS-PCR using sequence specific primers (forward primer for C allele: TCC TCA TAC TAA CCA TAT GAT CAA CAG TTC, forward primer for G allele: TCC TCA TAC TAA CCA TAT GAT CAA CAG TTG, internal control primer sequence: GAA GAT CAT ACC CGA AGT AGA GCT GC. For all forward primers a common reverse primer was used, with the sequence ATA CCA CAG TGA ACA TAA TTG TGC ATA CAT). The PCR was carried out in a thermal cycler with a total volume of 25 μL containing: 10X PCR Buffer, 3 mM MgCl_2_, 1 mg/mL nuclease free BSA, 50 pmol each of allele specific forward primer, reverse primer and internal control primer, 10 mM of each dNTP, 0.125 U *Taq* polymerase and 2 μl genomic DNA. The PCR cycle included an initial denaturation step of 5 min at 94 °C, followed by 35 cycles with denaturation for 30 s at 94 °C, annealing for 30 s at 59 °C elongation for 30 s at 72 °C and a final elongation of 10 min at 72 °C.

**Figure 1:**
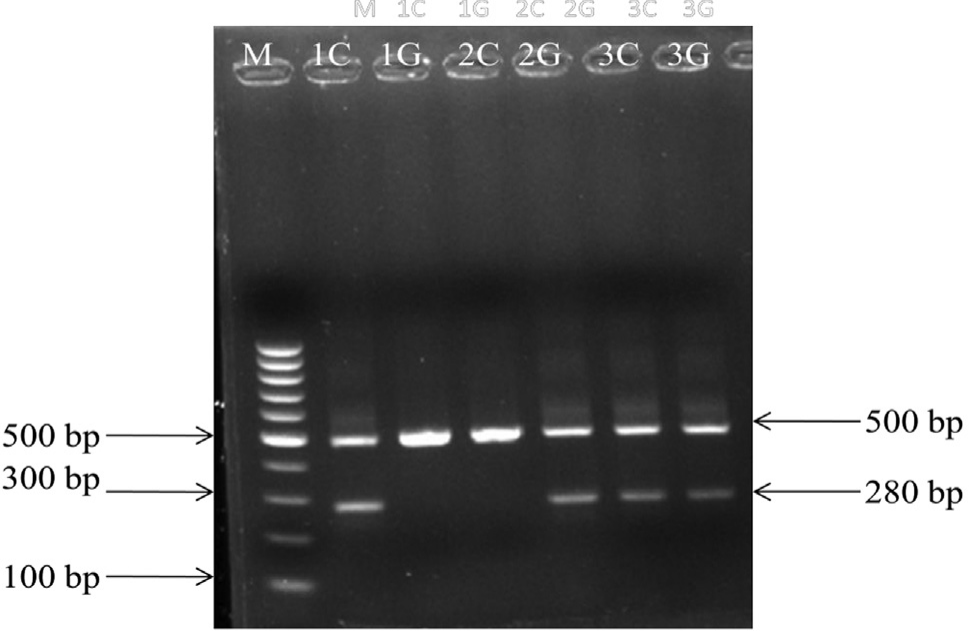
Allele specific ARMS-PCR products of *ANRIL* rs1333049 C/G polymorphism on 3% agarose gel. Lane M: 100 bp ladder, Lane 1C and 1G: homozygous wild CC genotype (500 and 280 bp), Lane 2C and 2G: homozygous mutant GG genotype (500 and 280 bp), Lane 3C and 3G: heterozygous CG genotype (500 and 280 bp)

Separate PCR was performed for both alleles of one SNP. The DNA fragments obtained were separated on 3% agarose gel stained with EtBr followed by visualization with UV transilluminator. A 280 bp fragment signified CC or GG genotype and 500 bp signified the internal control. If bands were seen for both alleles, it was interpreted as the heterozygous genotype (Figure 1). Correctness of genotyping was checked by re-genotyping of 10% of the samples. Results from the repeated samples were 100% consistent with our primary results.

#### Statistical analysis

Continuous variables that were not normally distributed were expressed as the mean ± standard deviation (SD). Categorical variables were reported as counts and percentages. Chi-square test was used to calculate the difference between baseline characteristics. To investigate the association of SNP and the susceptibility to CAD, multivariate logistic regression was applied adjusting for age and gender. Furthermore, recessive and dominant models were analysed. Stratified analysis for gender and age was also done for the assessment of association and expressed in odds ratio (OR) and 95% confidence interval (CI). A statistical significance of p *<* 0.05 was considered for the analysis. All the data was analysed using SPSS version 20.0 (SPSS, Inc., Chicago, IL) and Epi Info version 3.4.7 (CDC, Atlanta, GA).

### Results

The distribution of allele frequencies of the selected polymorphism followed the Hardy-Weinberg equilibrium. The baseline parameters of patients and controls are listed in Table 1. The results revealed a statistically significant variation between the two groups with respect to age, gender, smoking, drinking, waist to hip ratio, lifestyle, family history, dyslipidemia, diabetes, diet, hypertension, occupation, exercise, fasting blood sugar, uric acid, TC, VLDL, LDL, Apo A1, Apo B, but not with total lipids, triglycerides, BMI, HDL and hsCRP.

**Table 1:** Baseline characteristics of the studied population.

Phenotypic traits	Controls n (%)	Cases n (%)	p
**Age** (Mean ± SD; years)	50.95 ± 10.18	56.08 ± 9.55	< 0.001*
**Waist to hip ratio**	0.91 ± 0.08	0.97 ± 0.16	0.001*
**Blood Pressure**			
SBP (mmHg)			
	121.13 ± 9.92	133.67 ± 15.25	0.001*
DBP (mmHg)			
	80.35 ± 7.07	90.81 ± 13.45	0.046*
**Gender**			0.043*
Males	370(74)	397(79.4)	
Females	130(26)	103(20.6)	
**BMI**			0.069
Normal	270(54)	296(59.2)	
Obese	230(46)	204(40.8)	
**Smoking status**	Nil		< 0.001*
Non-smoker	327(65.4)		
Smoker	173(34.6)		
**Drinking status**	Nil		< 0.001*
Non-Drinker	348(69.6)		
Drinker	152(30.4)		
**Address**			0.342
Rural	233(46.6)	248(49.6)	
Urban	267(53.4)	252(50.4)	
**Family history**			< 0.001*
Nil	47(89.4)	304(60.8)	
+ve	53(10.6)	196(39.2)	
**Lifestyle**			< 0.001*
Active	431(86.2)	352(70.4)	
Sedentary	69(13.8)	148(29.6)	
**Diabetes**	Nil		< 0.001*
Negative	353(70.6)		
Positive	147(29.4)		
**Hypercholesterolemia**	Nil	500(100)	< 0.001*
Negative			
Positive			
**Hypertension**	Nil	500(100)	< 0.001*
Negative			
Positive			
**Diet**			0.001*
Veg	425(85)	385(77)	
Non veg	75(15)	115(23)	
**CK MB**	54.46 ± 45.81	34.36 ± 41.81	< 0.001*
**APO A1**	144.56 ± 26.41	121.09 ± 42.36	< 0.001*
**APO B**	98.67 ± 36.54	65.94 ± 23.77	< 0.001*
**hsCRP**	4.56 ± 0.20	2.35 ± 0.22	< 0.001*
**HDL-C**	52.35 ± 3.12	88.06 ± 9.11	0.673
**LDL-C**	135.65 ± 22.33	76.54 ± 32.50	0.005*
**VLDL-C**	48.57 ± 21.08	34.83 ± 20.74	0.043*
**FBG**	82.73 ± 19.30	111.56 ± 39.59	0.016*
**URIC ACID**	9.80 ± 3.45	6.26 ± 5.10	0.006*
**TC**	275.76 ± 53.49	144.95 ± 37.22	0.045*
**TRIGLYCERIDES**	198.23 ± 82.45	145.37 ± 65.62	0.468
**TL**	512.18 ± 116.05	436.05 ± 112.36	0.353

BMI: body mass index, SBP: systolic blood pressure, DBP: diastolic blood pressure, hsCRP: high-sensitivity C-reactive protein (hs-CRP), TC: total cholesterol, TG: triglycerides, HDL-C: high-density lipoprotein cholesterol, LDL-C: low density lipoprotein cholesterol; VLDL-C: very low density lipoprotein, FBG: fasting blood glucose, TL: total lipids. *=significant

Results revealed that the mutant (G) allele was more prevalent in patients (35.4%) than controls (33.8%) exhibiting a non-significant association to CAD with OR=0.930, 95% CI=0.77-1.13 and p=0.451 (Table 2).

**Table 2:** Genotypic and allelic frequencies of *ANRIL* rs1333049 C/G polymorphism in controls and cases.

Genotypes	Controls	Cases	Multiple logistic regression analysis	
	500(%)	500(%)	p	OR (95% CI)
*ANRIL* rs1333049 C/G				
CC	246(49.2)	166(33.2)	(ref.)	
CG	170(34)	314(62.8)	0.002*	2.883(1.475-5.638)
GG	84(16.8)	20(4)	< 0.001*	6.717(3.444-13.102)
**Alleles** C	662(66.2)	646(64.6)	(ref.)	
G	338(33.8)	354(35.4)	0.451	0.930(0.77-1.13)
**Dominant Model**				
CC	246(49.2)	166(33.2)	(ref.)	
CG+GG	254(50.8)	334(66.8)	0.004*	0.582(0.402-0.842)
**Recessive Model**				
CG+CC	416(83.2)	480(96)	(ref.)	
GG	84(16.8)	20(4)	< 0.001*	4.609(2.431-8.741)

ref.= reference; OR= odds ratio; CI= confidence interval; %= frequency; *= significant

The genotypic frequencies revealed the wild (CC) genotype to have a higher frequency in control (49.2%) than in CAD patients (33.2%). The heterozygous genotype (CG) was found to be highly prevalent among the cases (62.8%) in comparison to the controls (34.0%) with OR=2.883, 95% CI=1.475-5.638, p=0.002 and the homozygous mutant (GG) genotype had a higher frequency in controls (16.8%) than in the cases (4.0%) thereby conferring an increased risk with a high significance p < 0.001, OR=6.717 and 95% CI=3.444-13.102 (Table 2). The dominant and recessive models were also analysed to see the association of the polymorphism with CAD. A protective association was seen in the dominant model (OR=0.582, 95% CI=0.402-0.842 and p=0.004) whereas elevated risk association with CAD was observed in the recessive model (OR=4.609, 95% CI=2.431-8.741 and p < 0.001).

The data was stratified on the basis of age *i.e.* below 40 years and above 40 years (Table 3). High risk association in subjects above 40 years was documented for both the heterozygous and the mutant genotypes, with OR=2.647, 95% CI=1.287-5.447 and p=0.008 and OR=5.506, 95% CI=2.688-11.278 and p < 0.001 respectively. Also the G allele showed risk association (OR=1.600, 95% CI=1.226-2.088 and p < 0.001). For the age group less than 40 years, there is no mutant GG genotype in the cases. Therefore, calculations regarding allelic frequencies were not possible because one value is entirely missing. Nevertheless, a non-significant association could be seen in subjects below 40 years of age with the selected polymorphism. Stratification of the data on the basis of gender was also done (Table 3). In males, risk association was seen for both the heterozygous and the mutant genotypes, with OR=2.683, 95% CI=1.193-6.034 and p=0.017 and OR=5.902, 95% CI=2.628-13.255 and p < 0.001 respectively, whereas for females, only the mutant GG genotype revealed risk association, with OR=9.248, 95% CI=2.666-32.077 and a highly significant p < 0.001. Obesity also showed high risk association in heterozygous state with OR=3.546, 95% CI=1.157-10.863 and p=0.027 and mutant genotype with OR=8.633, 95% CI=2.823-26.400 and p < 0.001 (Table 3). Our study also intended to look for the association of sedentary lifestyle as a risk factor for CAD. Highly significant risk association was seen in CG and GG genotypes with OR=2.913, 95% CI=1.390-6.105 and p=0.005 and OR=7.024, 95% CI=3.358-14.693 and p < 0.001 (Table 3). Positive family history also highlighted association with CAD risk with OR=5.805, 95% CI=0.373-8.726 and p=0.018 for the heterozygous genotype and OR=7.453, 95% CI=1.445-38.438 and p=0.016 for the mutant genotype (Table 3). No association of drinking with the polymorphism and CAD risk was seen. Nevertheless, strong risk association with both the hetero and mutant genotype was seen with smoking (OR=1.837, 95% CI=0.548-2.279 and p=0.011 and OR=2.092, 95% CI=0.377-3.161 and p=0.027 respectively). However, only the mutant GG genotype showed risk association with diabetes with OR=2.330, 95% CI=1.458-3.857 and p=0.006 (Table 4).

**Table 3:** Stratified analysis for different parameters and its association with *ANRIL* rs1333049 C/G polymorphism.

Genotype	AGE < 40 YEARS				AGE ≥ 40 YEARS			
	Controls 379 (%)	Cases 27 (%)	p	OR (95% CI)	Controls 121 (%)	Cases 473 (%)	p	OR(95% CI)
CC	193(50.9)	8(29.6)	(ref.)		53(43.8)	158(33.4)	(ref.)	
CG	121(31.9)	19(70.4)		Can’t b calculated	49(40.5)	295(62.4)	0.008*	2.647 (1.287-5.447)
GG	65(17.2)	0(0)		Can’t b calculated	19(15.7)	20(4.2)	< 0.001*	5.506 (2.688-11.278)
Allele C	507(66.8)	35(64.8)	(ref.)		155(64.5)	611(64.3)	(ref.)	
Allele G	251(33.2)	19(35.2)	0.751	0.912 (0.511-1.626)	136(56.5)	335(35.7)	< 0.001*	1.600 (1.226-2.088)
	**MALE**				**FEMALE**			
	**Controls** 370 (%)	**Cases** 397 (%)	p	OR (95% CI)	**Controls** 130 (%)	**Cases** 103 (%)	p	OR (95% CI)
CC	173(46.8)	135(34)	(ref.)		73(56.2)	31(30.1)	(ref.)	
CG	135(36.5)	246(62)	0.017*	2.683 (1.193-6.034)	35(26.9)	68(66)	0.062	3.312 (0.944-11.622)
GG	62(16.8)	16(4)	< 0.001*	5.902 (2.628-13.255)	22(16.9)	4(3.9)	< 0.001*	9.248 (2.666-32.077)
Allele C	481(65)	516(65.3)	(ref.)		181(69.6)	130(61.9)	(ref.)	
Allele G	259(35)	278(34.7)	0.862	0.983 (0.797-1.212)	79(30.4)	76(38.1)	0.138	0.746 (0.506-1.099)
	**NON-OBESE**				**OBESE**			
	**Controls** 270 (%)	**Cases** 296 (%)	p	OR (95% CI)	**Controls** 230 (%)	**Cases** 204 (%)	p	OR (95% CI)
CC	133(49.3)	96(32.4)	(ref.)		113(49.1)	70(34.3)	(ref.)	
CG	92(34.1)	186(62.8)	0.064	2.681 (1.136-6.327)	78(33.9)	128(62.7)	0.027*	3.546 (1.157-10.863)
GG	45(16.7)	14(4.7)	0.076	6.086 (2.590-14.304)	39(17)	6(2.9)	< 0.001*	8.633 (2.823-26.400)
Allele C	358(66.2)	378(64)	(ref.)		304(66)	268(65.3)		
Allele G	182(33.8)	214(36)	0.389	0.641 (0.390-1.053)	156(44)	140(34.7)	0.887	1.432 (0.603-3.401)
	**ACTIVE LIFESTYLE**				**SEDENTARY LIFESTYLE**			
**Genotypes**	**Controls** 69 (%)	**Cases** 148 (%)	p	OR(95% CI)	**Controls** 431 (%)	**Cases** 352 (%)	p	OR(95% CI)
CC	27(39.1)	50(33.8)	(ref.)		219(50.8)	116(33)	(ref.)	
CG	32(46.4)	94(63.5)	0.374	2.129 (0.402-11.274)	138(32)	220(62.5)	0.005*	2.913 (1.390-6.105)
GG	10(14.5)	4(2.7)	0.099	3.952 (0.772-20.423)	74(17.2)	16(4.5)	< 0.001*	7.024 (3.358-14.693)
Allele C	86(61.4)	194(64.6)	(ref.)		576(66.9)	452(64.5)	(ref.)	
Allele G	52(38.6)	102(35.4)	0.511	1.987 (0.750-5.267)	286(33.1)	252(35.5)	0.277	0.458 (0.080-2.603)
	**No Family History**				**Family History**			
	**Controls** 447 (%)	**Cases** 304 (%)	p	OR(95% CI)	**Controls** 53 (%)	**Cases** 196 (%)	p	OR(95% CI)
CC	213(47.7)	96(31.6)	(ref.)		33(62.3)	70(35.7)	(ref.)	
CG	159(35.6)	195(64.1)	0.069	2.830 (1.299-6.163)	11(20.8)	119(60.7)	0.018*	5.805 (0.373-8.726)
GG	75(16.8)	13(4.3)	0.055	6.502 (3.013-14.029)	9(17)	7(3.6)	0.016*	7.453 (1.445-38.438)
Allele C	585(65)	387(63.4)	(ref.)		77(70)	259(66.4)	(ref.)	
Allele G	309(35)	221(36.6)	0.479	0.925 (0.745-1.147)	29(30)	133(33.6)	0.200	0.733 (0.455-1.179)

**Table 4:** Association of drinking, smoking and diabetes with *ANRIL* rs1333049 C/G polymorphism.

Genotype	Non-Drinker 348 (%)	Drinker 152 (%)	p	OR (95% CI)
CC	118(33.9)	48(31.6)	(ref.)	
CG	216(62.1)	98(64.5)	0.708	0.926(0.615-1.394)
GG	14(4)	6(3.9)	0.740	1.20(0.414-3.477)
Allele C	452(21.8)	194(63.8)	(ref.)	
Allele G	244(78.2)	110(36.2)	0.740	0.768(0.453-1.300)
	**Non-Smoker 327 (%)**	**Smoker 173 (%)**	**p**	**OR (95% CI)**
CC	111(33.9)	55(31.8)	(ref.)	
CG	202(61.8)	112(64.7)	0.011*	1.837(0.548-2.279)
GG	14(4.3)	6(3.5)	0.027*	2.092(0.377-3.161)
Allele C	424(64.8)	222(64.2)	(ref.)	
Allele G	230(35.2)	124(35.8)	0.841	1.124(0.333-3.798)
	**Non-diabetic 353 (%)**	**Diabetic 147 (%)**	**p**	**OR (95% CI)**
CC	115(32.6)	51(34.7)	(ref.)	
CG	223(63.2)	91(61.9)	0.689	1.086(0.721-1.637)
GG	15(4.2)	5(3.4)	0.006*	2.330(1.458-3.857)
Allele C	453(64.1)	193(65.6)	(ref.)	
Allele G	253(35.6)	101(34.4)	0.654	1.067(0.921-2.211)

## Discussion

This study aimed to understand risk association of *ANRIL* rs1333049 C/G to CAD in a North Indian population. Sequence specific ARMS-PCR was used for genotyping and results showed a considerable risk association towards CAD and the same was observed in the recessive model (Table 1). Moreover, the allelic frequencies also conferred a significant association with CAD (p < 0.05). Also, the rs1333049 C/G polymorphism showed risk towards CAD for age above 40 years, males and females, obesity, sedentary lifestyle, family history, diabetes and smoking.

Numerous studies document ANRIL rs1333049 C/G polymorphism and its correlation with CAD risk and progression (Samani *et al.*, 2007, 2008; Welcome Trust Case Control Consortium, 2007; Schunkert *et al.*, 2008; Karvanen *et al.*, 2009; Muendlein *et al.*, 2009; Bressler *et al.*, 2010; Dandona *et al.*, 2010; Ellis *et al.*, 2010; McPherson, 2010; Palomaki *et al.*, 2010; Wang *et al.*, 2010; Mendonça *et al.*, 2011; O’Donnell *et al.*, 2011; Angelakopoulou *et al.*, 2012; Plichart *et al.*, 2012) and ischaemic stroke (Karvanen *et al.*, 2009; Smith *et al.*, 2009). The above mentioned studies majorly comprised of Caucasian descent populations.

A few studies from India have tried to explore genetic polymorphisms at this selected locus. A GWA study done with a South Indian population on 9p21 locus reported two SNPs (rs2383207 and rs10757278) conferring elevated risk to CAD (AshokKumar *et al.*, 2011). Also, the work done by Kumar *et al.* (2011) on the North Indian population, reports three SNPs (rs2383206, rs1333040 and rs10116277) at 9p21 locus to be associated with CAD risk. The rs10757278 polymorphism at the same locus also correlates with CAD risk as reported by two studies by (Maitra *et al.*, 2008; Bhanushali *et al.*, 2011). Only two studies report data on rs1333049 C/G and CAD risk in West and North Indian populations. (Bhanushali, *et al.*, 2013) recruited 229 CAD patients and 136 controls from West India and revealed an association towards CAD with an OR=2.460, 95% CI=1.139–5.314 and p=0.022). Kashyap *et al.* (2018) in their study on North Indian population reported risk for both the allelic and genotypic frequencies. This study also showed an association with CAD with an OR=6.717, 95% CI=3.444-13.102 and p < 0.001. Thus, the results point towards the fact that both the North Indians as well as the West Indians are susceptible to CAD due to this polymorphic change in *ANRIL* rs1333049.

The multiple conventional risk factors for CAD such as diabetes, hypertension, dyslipidemia, *etc.* become additive with increasing age, thereby contributing to atherosclerosis leading to CAD. A positive risk association in the subjects above 40 years of age was seen in the study with the mutant genotype having an OR=5.506 with a highly significant p < 0.001 (Table 2). However, Bhanushali *et al.* (2013) reported the SNP to be robustly associated with premature or the early onset CAD which is also supported by the results of Meng *et al.* (2008), Ellis *et al.* (2010) and the association was supported by meta-analysis study done by Palomaki *et al.* (2010). But in present study, no mutant in the CAD patients was found below 40 years of age in the pre-specified sub-group analysis based on age. The overall frequency of GG mutant genotype in our selected population is 10.4% *i.e.* only 104 individuals have the GG genotype thereby pointing towards its low prevalence in our selected North Indian population.

The discrepancy in results emphasizes the need to genotype all the risk variants particularly at this locus, as this will help in delineating the varied risk associations in different populations to CAD. However, the impact of the polymorphism with disease extent and severity is disputable with Ye *et al.* (2008) and Dandona *et al.* (2010) stating it as a predictor of severity, whereas Anderson *et al.* (2008) and Chen *et al.* (2009) contradicting it. Additionally, the present study results showed a strong association with the family history which is in accordance with previous work (Preuss *et al.*, 2010; Scheffold *et al.*, 2011). Gender stratified analysis depicted significant association in both the genders which is in harmony to the results reported by Ahmed *et al.* (2013) on Northern Pakistani population.

In summary, we conclude that *ANRIL* rs1333049 C/G is associated with susceptibility to CAD in North Indian population and also associations with many risk factors have been documented. Although 9p21 locus association with risk of CAD is very well recognized, relationship with the clinical outcomes remains unclear and unanswered. The chosen SNP is intronically located but still can affect gene expression. Therefore, future studies with higher sample size, multiple SNPs from the locus and linkage studies are needed to authenticate our results that might cause identification of more SNPs at this particular locus as biomarkers for CAD predisposition.

Analyzing the SNPs which are substantially associated with CAD in North Indian population will be useful to identify promising SNP-CAD associations unique to the population. Moreover, CAD poses threat not only to an individual and his family but also to the community and the nation on the whole as the most productive years of one’s life is spent struggling with the disease. The drastic change in lifestyle and eating habits and the increased tendency to rely on machines and other forms of assistance has substantially decreased one’s physical effort and rendered individuals highly susceptible to CAD. Comprehending the genetic foundation of CAD is highly needed these days that will help in screening individuals at high risk and will also lay the groundwork for the coacervation of genetic data and routine clinical practice, which can one day spearhead the arena of “personalized medicine”.
